# Inferring eye position from populations of lateral intraparietal neurons

**DOI:** 10.7554/eLife.02813

**Published:** 2014-05-20

**Authors:** Arnulf BA Graf, Richard A Andersen

**Affiliations:** 1 Division of Biology and Biological Engineering, California Institute of Technology, Pasadena, United States; Universidad Nacional Autonoma de Mexico, Mexico

**Keywords:** non-human primate, oculomotor system, population decoding, Bayesian inference, other

## Abstract

Understanding how the brain computes eye position is essential to unraveling high-level visual functions such as eye movement planning, coordinate transformations and stability of spatial awareness. The lateral intraparietal area (LIP) is essential for this process. However, despite decades of research, its contribution to the eye position signal remains controversial. LIP neurons have recently been reported to inaccurately represent eye position during a saccadic eye movement, and to be too slow to support a role in high-level visual functions. We addressed this issue by predicting eye position and saccade direction from the responses of populations of LIP neurons. We found that both signals were accurately predicted before, during and after a saccade. Also, the dynamics of these signals support their contribution to visual functions. These findings provide a principled understanding of the coding of information in populations of neurons within an important node of the cortical network for visual-motor behaviors.

**DOI:** http://dx.doi.org/10.7554/eLife.02813.001

## eLife digest

Whenever we reach towards an object, we automatically use visual information to guide our movements and make any adjustments required. Visual feedback helps us to learn new motor skills, and ensures that our physical view of the world remains stable despite the fact that every eye movement causes the image on the retina to shift dramatically. However, such visual feedback is only useful because it can be compared with information on the position of the eyes, which is stored by the brain at all times.

It is thought that one important structure where information on eye position is stored is an area towards the back of the brain called the lateral intraparietal cortex, but the exact contribution of this region has long been controversial. Graf and Andersen have now clarified the role of this area by studying monkeys as they performed an eye-movement task.

Rhesus monkeys were trained to fixate on a particular location on a grid. A visual target was then flashed up briefly in another location and, after a short delay, the monkeys moved their eyes to the new location to earn a reward. As the monkeys performed the task, a group of electrodes recorded signals from multiple neurons within the lateral intraparietal cortex. This meant that Graf and Andersen could compare the neuronal responses of populations of neurons before, during, and after the movement.

By studying neural populations, it was possible to accurately predict the direction in which a monkey was about to move his eyes, and also the initial and final eye positions. After a movement had occurred, the neurons also signaled the direction in which the monkey's eyes had been facing beforehand. Thus, the lateral intraparietal area stores both retrospective and forward-looking information about eye position and movement.

The work of Graf and Andersen confirms that the LIP has a central role in eye movement functions, and also contributes more generally to our understanding of how behaviors are encoded at the level of populations of neurons. Such information could ultimately aid the development of neural prostheses to help patients with paralysis resulting from injury or neurodegeneration.

**DOI:** http://dx.doi.org/10.7554/eLife.02813.002

While many visual brain areas contain representations of the current eye position, the nature and the role of this eye position signal (EPS) are still unclear. This is surprising because the EPS is essential for our ability to perceive and interact with the world around us. For instance, the EPS combined with the visual input falling on the retina allows us to perceive the world as stable even though we move our eyes (Helmholtz, 1867). The EPS also plays a role in accurate visually guided movements (Zipser and Andersen, 1988; Salinas and Abbott, 1996; Pouget et al., 2002) and motor learning (Lewis et al., 1994). The lateral intraparietal area (LIP) is one of the areas representing the EPS (Andersen et al., 1985; Andersen et al., 1987; Gnadt and Andersen, 1988; Andersen et al., 1990; Schall and Thompson, 1999; Goldberg et al., 2002). However recent studies have reported that the EPS is too slow to support the functions of spatial stability and coordinate transformations (Xu et al., 2012), or is inaccurate around the time of saccadic eye movement (Morris et al., 2012; Morris et al., 2013). Here we show that when quantified across neuronal populations and a wide range of oculomotor behaviors, the EPS in LIP is accurate before, during, and after eye movements. Contrary to previous reports, the dynamics of the EPS are consistent with LIP supporting the aforementioned sensorimotor functions.

**Figure 1.**
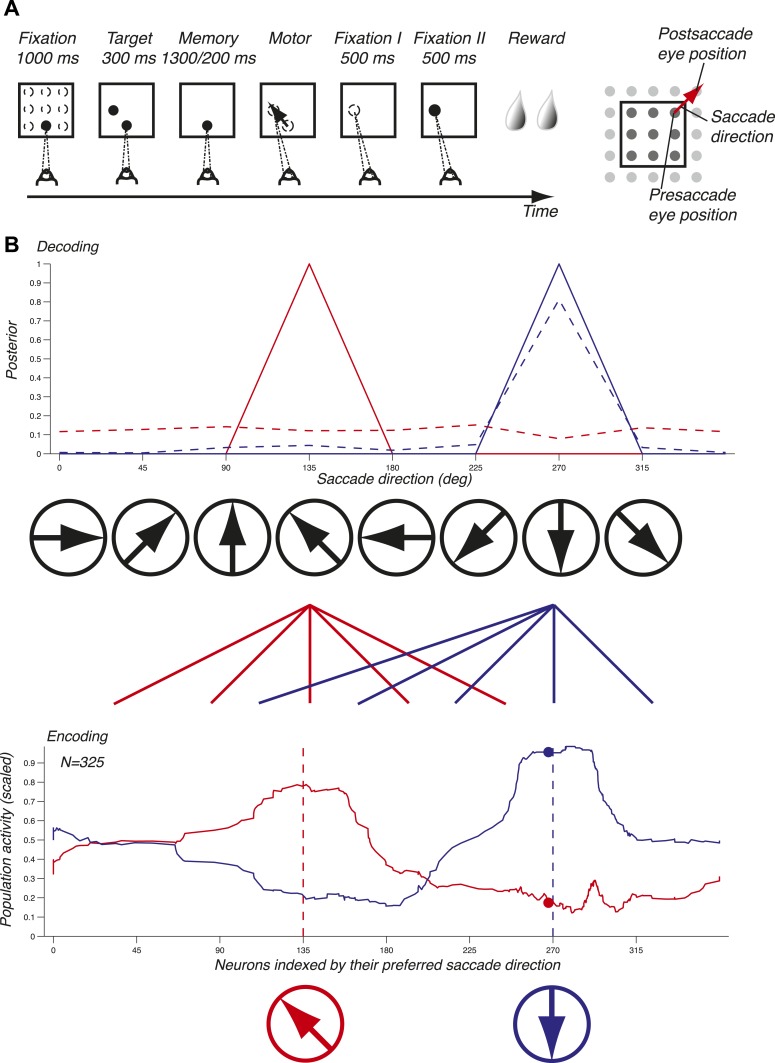
Task and decoding mechanisms. (**A**) Scheme of the task and the three oculomotor behaviors. (**B**) Predicting saccade direction from the population activity in LIP. The population activity of 325 neurons (red and blue curves, bottom panel) during the memory epoch was computed at the preferred eye position (derived from the fixation epoch) for two planned saccade directions (180 and 270 deg, dotted lines, bottom panel), and yielded the prediction (red and blue curves, top panel). The responses of a single neuron (red and blue dots, bottom panel) yielded the dotted probability distributions (top panel). **DOI:** http://dx.doi.org/10.7554/eLife.02813.003

We used a delayed memory saccade task on a grid that exhaustively samples pre- and postsaccade eye positions and saccade directions, thus yielding a complete tiling of the spatial variables (Figure 1A and ‘Materials and methods’). Presaccade eye position and saccade direction were by design two independent variables. We recorded 51 ensembles of single units, for a total of 343 neurons, in LIP in two non-human primates (‘Materials and methods’).

To illustrate the mechanisms of predicting oculomotor behaviors from neuronal populations, we computed the population activity in the memory epoch for two planned saccade directions (135 and 270 deg) starting at the preferred eye position (red and blue curves, bottom panel, Figure 1B). The population response for each saccade direction was bell-shaped and centered on the neurons that most vigorously responded for the planned saccade. We subsequently inferred the planned saccade direction based solely on these two population activities by computing the probability of a saccade direction given the observed population response (solid lines, red for 135 deg and blue for 270 deg, top panel, Figure 1B). Each population activity perfectly predicted (i.e., with a probability of 1) the planned saccade direction underlying it. We then asked how well saccade directions could be predicted using only one neuron instead of the entire population: the dots in bottom panel represent the responses of this neuron to the two saccade directions, and the dotted lines in the top panel show the corresponding prediction probabilities. We found that while one planned saccade direction could be well, albeit not perfectly, predicted (270 deg, blue dotted line), the other direction (135 deg, red dotted line) could not be predicted (almost uniform distribution). Predictions based on the population activity, however, were informative across all saccade directions, and could thus potentially reveal properties that were hidden at the level of single neurons. The importance of a population representation for inference is further reinforced in LIP because it contains an abundance of simultaneously expressed signals, for example eye position and saccade direction. Surprisingly, LIP has to our knowledge not yet been studied at the population level using simultaneous neuronal recordings or an exhaustive sampling of saccade directions and eye positions.

**Figure 2.**
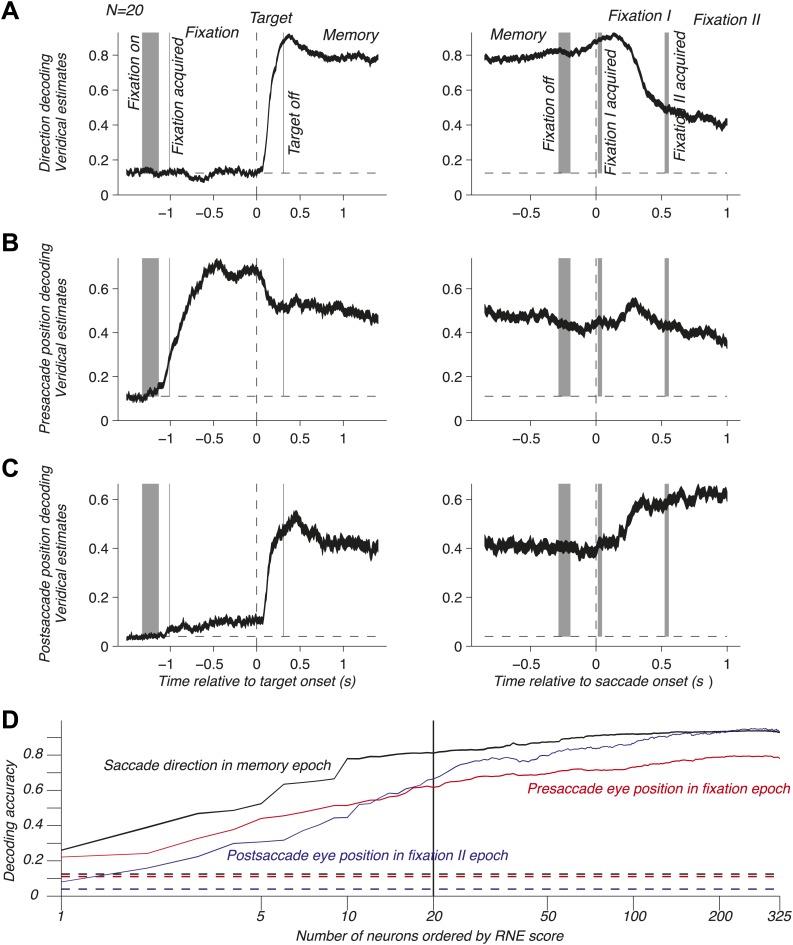
NPC across the task for one population and as function of the number of neurons. (**A**–**C**) Time course of the NPC for a population of 20 independently recorded neurons for the three oculomotor behaviors. The dark curves are the decoding accuracies (mean ± SEM across trials) with spike times aligned to the target onset (left column) and saccade onset (right column). The horizontal dotted lines represent chance levels (1/8, 1/9 and 1/25 respectively). The shaded gray areas (mean ± SD) represent the different task events: fixation on, fixation acquired, target off (left column) and fixation off, fixation I acquired, fixation II acquired (right column). (**D**) NPC for each of the three oculomotor behaviors during different task epochs as function of the number of neurons ordered by increasing importance for the NPC (mean ± SEM). **DOI:** http://dx.doi.org/10.7554/eLife.02813.004

To address population coding in LIP, we used Bayesian inference (Dayan and Abbott, 2001; Jaynes, 2003; Pouget et al., 2003; Graf et al., 2011) to compute the neuronal population code (NPC): the accuracy with which pre- and postsaccade eye positions and the saccade direction could be predicted from the population response. For this, we assumed that the neurons were independent and that the response statistics of each neuron followed a Poisson distribution (‘Materials and methods’). We computed the time course of the NPC for the three oculomotor variables across the entire task using a population of 20 independently recorded neurons. These neurons were selected to illustrate the contribution of LIP to mediating eye position and movement information (see below). The NPC of saccade direction (Figure 2A) was at chance during fixation, then strongly increased during the visual epoch (transient sensory response), and finally reached a sustained level during the memory epoch. It was best predicted right after the saccade and subsequently decayed. These findings indicate that the NPC contains a prospective component consistent with the well-documented saccade planning signal (Gnadt and Andersen, 1988; Snyder et al., 1997) and a retrospective component that, albeit only available for a limited time, could be used for learning or monitoring of recent saccades. Surprisingly, the presaccade eye position was coded similarly well before and after the saccade (Figure 2B), despite a new postsaccade eye position being acquired, effectively transitioning the EPS from current state to past state. The long lasting component after the saccade is consistent with a memory of where the eye had been and may be useful for learning and calibration at a longer time scale than for the saccade direction signal. The NPC of the postsaccade eye position (Figure 2C) had a more intricate nature because it is the combination of the presaccade eye position and saccade direction signals (‘Materials and methods’). As such, it was predictive of the postsaccade position even during the fixation epoch, and its accuracy strongly increased when the saccade direction information was made available (target onset). The NPC anticipated the future eye position long before the saccade, thus showing a prospective coding. This prediction may result from the simultaneous representation of both the presaccade eye position and the planned eye movement direction after the saccade target onset. The NPC was most accurate when the postsaccade eye position was realized, effectively transitioning from a predictive presaccade state to an updated current postsaccade state.

The neurons composing the population in Figure 2A–C were not empirical samples collected simultaneously: they were a collection of the 20 independently recorded neurons that were most relevant for the NPC (‘Materials and methods’). The NPC increased dramatically for small populations of such ‘optimized’ neurons and subsequently flattened out (Figure 2D). This result suggests that the NPC in LIP is sparse: a few carefully selected neurons do nearly as well as many more, and 20 neurons represent a good tradeoff between population size and prediction accuracy.

**Figure 3.**
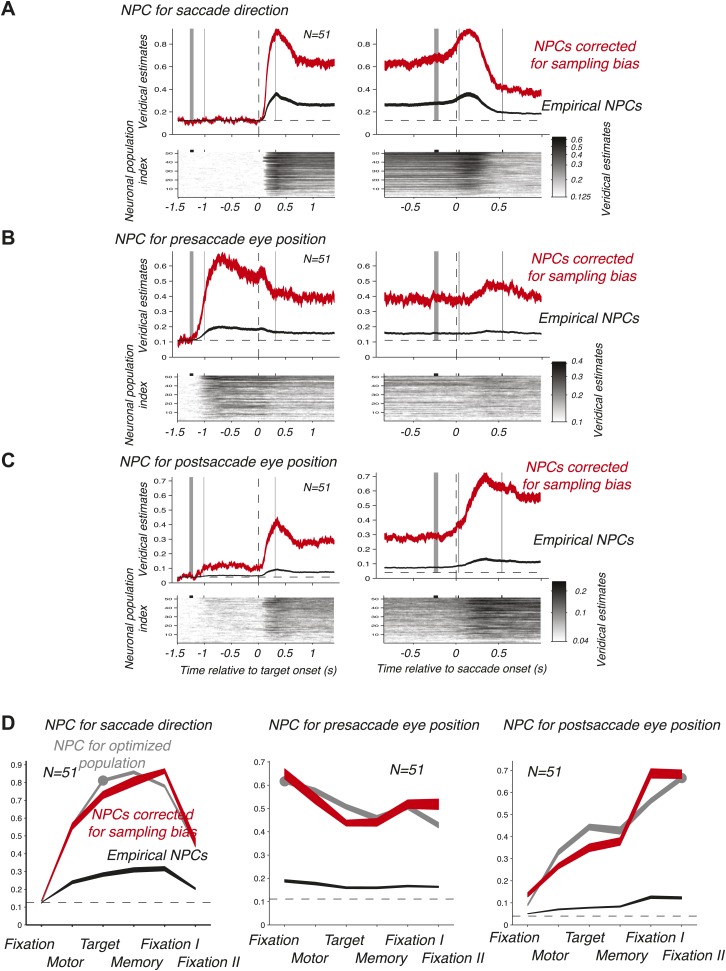
Time course of the NPC. (**A**–**C**) Time course of the NPC for 51 empirical samples of simultaneously recorded neurons for the three oculomotor behaviors. The images (bottom panels) represent the NPC with spike times aligned to the target onset (left column) and saccade onset (right column). Each horizontal slice represents the NPC of one population. The dark curves in the upper panels are the time courses of the NPCs averaged across populations (mean ± SD), and the red curves the sampling-corrected NPCs using the same conventions as in Figure 2.(**D**) Accuracy of the NPC for each behavior in each task epoch. The black curves are the accuracies averaged across empirical populations of simultaneously recorded neurons (mean ± SEM), and the red curves are the sampling corrected empirical populations (mean ± SEM), and the gray curves are the accuracies of the population of 20 ‘optimized’ neurons (mean ± SEM, the dots indicating the epochs used in Figure 2D). **DOI:** http://dx.doi.org/10.7554/eLife.02813.005

We then computed the time course of the NPC for each of the 51 empirical populations of simultaneously recorded neurons for the three oculomotor behaviors. These individual time courses (images in the bottom panels of Figure 3A–C) were consistent across populations. We therefore averaged the NPC across populations (black traces in the upper panels of Figure 3A–C), and found that the magnitude of these traces were significantly lower than for the NPC of the ‘optimized’ population of Figure 2A–C. This finding is readily explained considering that the empirical populations were subject to sampling bias, for example incomplete tiling of the parameter space (Graf et al., 2011), this bias being avoided by construction of the ‘optimized’ pool of neurons. More germane to this research, the time course of the NPCs had similar dynamic signatures to the ‘optimized’ population. This was best visualized when looking at the average bias-corrected NPC where the NPC of each population was first scaled to match on average the NPC of the ‘optimized’ population (red traces in the upper panels of Figure 3A–C, ‘Materials and methods’). We corroborated these findings by computing the average decoding accuracy across the different task epochs (Figure 3D) using the root-mean square of the NPC time course (‘Materials and methods’).

We have shown that the EPS is accurately carried by populations of LIP neurons before, during and after an eye movement. We next investigated the dynamics of this signal, and asked whether they were consistent with the three hypothesized functions of the EPS. We computed the onsets and peaks of the time course of the NPC (‘Materials and methods’) of empirical populations of simultaneously recorded neurons, and found that the three oculomotor signals drastically differed in their temporal signatures.

**Figure 4.**
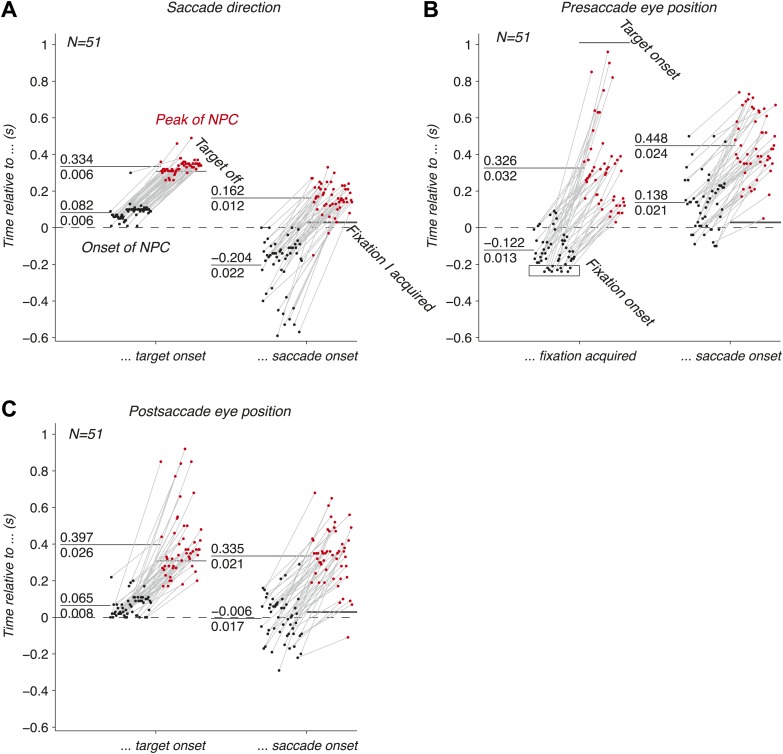
Dynamics of the NPC. (**A**–**C**) Timing of the onsets (black dots) and peaks (red dots) of the NPC across empirical populations of simultaneously recorded neurons for the three oculomotor behaviors (times spread out along abscissa for visualization). **DOI:** http://dx.doi.org/10.7554/eLife.02813.006

The onset of the NPC for the saccade direction (Figure 4A) right after the target onset was similar to the typical visual response latencies of neurons (Schmolesky et al., 1998). A small fraction of onsets happened earlier, and were attributed to noise in the estimation of the NPC time course and computation of the onsets. The NPC of the saccade execution signals rose before the saccade, consistent with the peri-saccadic burst of activity that begins before the eye movement (Andersen et al., 1987). This result suggests that populations of LIP neurons play a role in perceptual stability and state estimation (Mulliken et al., 2008). It also indicates that the NPC might be initiated by a corollary discharge signal, an internal signal monitoring neuronal movement commands (Sommer and Wurtz, 2002). The peak of the NPC occurred after the saccade, indicating that the full strength of the NPC for saccade direction also includes signals of proprioceptive origin. The corollary discharge of eye movements (e.g., saccades) could potentially originate in FEF or another motor area (Sommer and Wurtz, 2002), whereas the proprioceptive signals (e.g., eye muscle proprioceptors) could originate in area 3a (Wang et al., 2007).

The EPS of the presaccade current eye position appeared before the initial fixation was acquired (Figure 4B), suggesting a corollary discharge predicting the future position of the eye in the presence of a visual input. This EPS transitioned from current to past state with a temporal delay in the absence of visual input and peaked well after the saccade (Figure 4B). The NPC of the presaccade eye position close to the saccade did not allow us to trace its origins because it occurred long after the presaccade eye position was acquired.

The EPS of the postsaccade eye position, computed using the current eye position and the saccade direction signals, was first strongly revealed right after the target onset with timing consistent with the NPC of the saccade direction signal (Figure 4C). The NPC of the postsaccade eye position strongly increased around the saccade onset, thus transitioning from a predictive to a current state. In some cases the signal rose before, and some cases after the saccade. These findings suggest that the EPS at the saccade onset has corollary discharge and proprioceptive components respectively. This result contrasts with the presaccade signal that began before the fixation was acquired (Figure 4B). We hypothesize that this difference is due to the multiplicative effect (gain field) of a visual inputs (fixation light) and eye position that may lower the threshold for cell activity, thus producing an earlier expression of the presaccade eye position signal. The peak of the NPC occurred after the saccade, consistent with a proprioceptive contribution. Contrary to recent findings (Xu et al., 2012), our results show that the EPS was fast enough to represent eye position, in fact being very prospective, and that gain fields are updated almost instantly.

The results of this study show that the EPS is accurate throughout the course of an eye movement even long before and long after the saccade and has rapid transients that allow it to accurately reflect oculomotor behaviors. These findings favor the three hypothesized roles of the EPS. First, visual perceptual stability requires an EPS that is fast, as corroborated by our findings. We however also found that the EPS, at least in LIP, does not reflect visual mislocalization around the saccade (the accuracy of the NPC does not decrease around the saccade), thus suggesting that this might take place in another visual-motor area. Second, we found that the EPS can have a central function for coordinate transformations because the EPS was fast and present immediately before and after the eye movement. The EPS is ideally suited for state estimation because it is composed of sensory feedback (proprioception) and forward model (corollary discharge) signals. It can thus give the best estimate of the current state of the eye and is also consistent with the ‘remapping’ seen across eye movements (Duhamel et al., 1992; Vaziri et al., 2006). Third, we showed that the EPS could be used as a calibration signal for learning and adapting eye movements because it occurred immediately after the saccade and was maintained for a long period of time.

Our findings show that the timing of information of the EPS is consistent with the hypothesized roles of LIP in visual-motor behaviors (Andersen et al., 1985). These results strengthen further the central role of LIP for oculomotor behaviors despite also having high-level cognitive functions (Duhamel et al., 1992; Shadlen and Newsome, 1996; Snyder et al., 1997; Platt and Glimcher, 1999; Andersen and Buneo, 2002; Corbetta and Shulman, 2002; Bisley and Goldberg, 2003; Freedman and Assad, 2006). Moreover, the findings provide a better understanding of the fundamental concepts of how information is processed in the cerebral cortex at the level of populations of neurons. The study of the NPC can guide the development of brain-controlled neural prosthetics that can assist paralyzed patients with limb and oculomotor paralysis resulting from traumatic accidents, peripheral neuropathies, amyotrophic lateral sclerosis, multiple sclerosis, and stroke.

## Materials and methods

### Delayed memory saccade task on a grid

The animals performed delayed memory saccades on a Cartesian grid whose nodes were spaced by 8 deg. The animals maintained fixation for 1000 ms at one randomly chosen location on the 3 × 3 grid. Next a target was flashed for 300 ms in one location randomly chosen from the eight neighboring locations, and the animals were required to maintain fixation during this epoch. The animals had to remember the target location for 1300 ms ± 200 ms (drawn from a uniform distribution). Upon extinction of the fixation, they made a saccade to the remembered target location in complete darkness, thus acquiring a postsaccade fixation on a 5 × 5 grid. They maintained fixation there for 500 ms (fixation I) in the absence of visual input. A fixation light was subsequently turned on at the target location for another 500 ms, possibly triggering a small corrective saccade. Upon completion of this second fixation (fixation II), the stimulus was removed and the animals were rewarded by a drop of water. We only considered correct trials. The animals completed ∼11 random blocks, for a total of ∼11 × ((3 × 3) × 8) = 792 saccades. The eye position was tracked by an infrared camera, and the signal was sampled at 2 kHz (EyeLink, SR Research, Ontario, Canada). All behavioral variables were instructed and monitored in real-time (LabView, National Instruments, Austin, TX, USA).

The delayed memory task allowed us to examine separately neuronal signals mediating sensory inputs (vision), planning, and motor commands. Furthermore, we were able to study how pre- and postsaccade eye positions and saccade directions are represented because we extended the delayed memory task to cover a grid. One novelty of this task is that eye position and saccade directions were sampled exhaustively on a Cartesian grid, thus yielding a complete set of independent oculomotor variables. In contrast to previous single neurons studies where behaviors were tailored to best drive a given neuron (e.g., preferred and anti-preferred saccade direction), another novelty of the task resides in the fact that we used a fixed and finite set of oculomotor behaviors to drive neuronal populations as a whole.

### Neuronal population recordings

Two adult male rhesus monkeys (*Macaca mulatta*, monkey S 14.3 kg and monkey P 13.7 kg) were used in the experiments. All procedures were in accordance with the guidelines of the Caltech Institutional Animal Care and Use Committee and the National Institute of Health *Guide for the Care and Use of Laboratory Animals*. Each animal was implanted with a headpost and custom-made recording chamber under aseptic conditions using isoflurane anesthesia. Both animals received postoperative analgesics during postsurgical recovery. We used MRI scans as guides for the location of LIP.

We recorded populations of LIP neurons using a 5-channel microdrive with movable quartz-platinum/tungsten microelectrodes (Thomas Recording, Giessen, Germany). The neuronal data were filtered between 100 Hz and 8 kHz (preamplifier, Plexon, Dallas, TX, USA), and then sampled at 40 kHz (Multichannel Acquisition Processor, Plexon). Single units were sorted online using a dual-window discriminator based on the shape of the waveform. At the beginning of each recording session, we verified that each electrode tip was located in LIP by obtaining a vigorous sensory, planning and motor activity during a center-out delayed memory saccade task. Each animal was recorded in a different hemisphere. Hence, when combining the neuronal populations recorded in both animals, we were able to overcome the component of the sampling bias associated with the fact that LIP has contralateral response fields for visual stimuli and movement direction (Quian Quiroga et al., 2006). To empirically quantify the component of the sampling inherent to the homogeneity of neurons in the sample, we acquired a different population of simultaneously recorded neurons for each session.

We gathered empirical samples of simultaneously recorded neurons for three reasons. First, this allowed us to assess the sampling bias underlying the computation of the NPC. Second, the neurons recorded simultaneously were subject to exactly the same experimental conditions, thus eliminating session-to-session variability from the computation of the NPC. Third, simultaneously recorded neurons gave a more natural sample of the NPC because these neurons were involved—although to different degrees—in exactly the same behaviors within a session and across sessions. This is in stark contrast to most single neuron studies where the task was optimized to best drive the studied neuron.

### Computing the accuracy of the neuronal population code

To address population coding in LIP, we studied the accuracy with which pre- and postsaccade eye positions and the saccade direction could be predicted from the population response. This neuronal population code (NPC) is a single summary metric that encompasses the response properties of multiple neurons across a range of oculomotor behaviors, unlike most classical measures of single-cell electrophysiology where each neuron and behavior are studied separately. The NPC describes the accuracy of cortical computations relevant to a given task within an area, and the information available in principle to a downstream neuron that gets synaptic inputs from this population.

We used Bayesian inference to model the NPC by predicting the accuracy with which a behavior can be inferred from the population response taken to be the spike count of each neuron over a given temporal window (Foldiak, 1993; Seung and Sompolinsky, 1993; Salinas and Abbott, 1994; Sanger, 1996; Oram et al., 1998; Dayan and Abbott, 2001; Jaynes, 2003; Pouget et al., 2003; Sanger, 2003; Averbeck and Lee, 2004; Brown et al., 2004; Ma et al., 2006; Graf et al., 2011). The prior *p(b)* on the behavior *b*, i.e. the probability of a given behavior, was computed from the number of occurrences of that behavior. It represents the behavioral history, and is entirely defined by the task (the task has no error trials). The likelihood function *p(****r****|b)* is the probability to obtain a population response (spike count) ***r*** given a behavior *b*, evaluated across behaviors. It is a description of the encoding process that addresses how a behavior is represented at the level of neuronal populations. We computed the likelihood by assuming that the *N* neurons were independent, yielding:

$$p\left(r|b\right)=\overset{N}{\underset{i=1}{\prod }}p\left(r_{i}|b\right)$$

Next, we used a parametric approximation for the likelihood function of each neuron. The simplest approximation is to assume that the spike counts of a neuron for a given behavior follow a Poisson distribution:

$$p\left(r|b\right)=\frac{\mu {\left(b\right)}^{r}}{r!}\text{exp}\left(-\mu \left(b\right)\right)$$

where μ(b) is the mean response across all trials corresponding to the behavior *b*. This model requires the empirical determination of one parameter: the mean response (tuning curve). This model has been widely used, and was also extended to take correlations between neurons into account (Sanger, 1996; Oram et al., 1998; Ma et al., 2006; Graf et al., 2011). Alternatively, a more complicated approximation is to assume that the spike count distribution can be approximated by a truncated Gaussian over positive integers:

$$p\left(r|b\right)=\frac{G\left(r,\mu ,\sigma ,b\right)}{\overset{\infty }{\underset{0}{\int }}G\left(r,\mu ,\sigma ,b\right)dr}$$

where: $G\left(r,\mu ,\sigma ,b\right)=\frac{1}{\sqrt{2\pi \sigma {\left(b\right)}^{2}}}\text{exp}\left(-\frac{{\left(r-\mu \left(b\right)\right)}^{2}}{2\sigma {\left(b\right)}^{2}}\right)$

This model requires the empirical determination of two parameters: the mean μ(b) and the variance $\sigma {\left(b\right)}^{2}$ of the response across all trials corresponding to the behavior *b*. Both approximations gave decoding accuracies (see below) that were strongly correlated (r^2^ = 0.95, p=0).

The posterior function p(b|r) is the hallmark of decoding: it is the probability to obtain a behavior given a neuronal population response. By Bayes' theorem, it is proportional to the product of the likelihood function and the prior:

$$p\left(b|r\right)=\frac{p\left(r|b\right)p\left(b\right)}{p\left(r\right)}$$

where p(r) is the partition function. In order to ensure good generalization (and thus avoid overfitting), we evaluated the posterior function using a leave-one-out cross-validation scheme (Duda et al., 2001). In other words, the trials that were used to evaluate the posterior were different from the trials used to compute the parameters of the model.

The posterior function is a probability distribution that informs us how likely a behavior can be associated with a given neuronal population response. We considered the maximum of the posterior distribution as the estimate corresponding to a population activity (MAP estimate). We determined the prediction accuracy of each behavior by computing the proportion of veridical MAP estimates evaluated across all trials, i.e., the fraction of trials where true and estimated behaviors coincided (exact estimates). Finally, the accuracy of the NPC was the average of the prediction accuracy of each behavior. The mean and standard error of the NPC were determined using 1000 bootstraps across all trials.

Our task allowed us to study three oculomotor behaviors: the 3 × 3 = 9 presaccade eye positions, the eight saccade directions and the 5 × 5 = 25 postsaccade (future) eye positions. These behaviors were entangled in a single population response. In each trial, the animal executed one out of a total of 9 × 8 = 72 oculomotor behaviors. Each population response thus predicted one saccade direction and one presaccade eye position. Because presaccade eye position and saccade direction were independent, their respective NPCs were computed separately, for example the NPC for the saccade direction was determined using the fraction of correct saccade direction estimates across all trials. The postsaccade eye position was the vector sum of the presaccade eye position and the saccade direction. Multiple presaccade eye positions and saccade directions could yield the same postsaccade eye position. The postsaccade eye position was thus dependent on the presaccade eye position and the saccade direction. We explicitly modeled this dependency in the Bayesian framework by translating the vector sum of the behaviors into a marginalization of the posterior distributions: the posterior of the postsaccade eye position was the sum across the posteriors of all presaccade eye positions and saccade directions that yielded the same postsaccade eye position. In other words, the vector sum of the behaviors translated into a sum of their respective posteriors.

### Time course of the accuracy of the NPC

To determine the time course of the NPC, we first aligned the spike times for each trial to either the target onset or the saccade onset. The saccade onset (reaction time) was determined offline based on the saccade velocity using a method adapted from (Engbert and Kliegl, 2003). For each neuron, we subsequently computed the spike counts over causal boxcar windows (rectangular windows looking only in the past) of length 250 ms sliding in 10 ms steps. We chose a length of 250 ms because this interval is short enough compared to the length of the epochs in the task, but long enough to have sufficient spike counts for decoding. For each time step, we then computed the prediction accuracy (mean ± SEM from bootstrap estimates), yielding the time course of the NPC. The inference was thus done on intervals of identical length (250 ms) across the entire task.

To compute the decoding accuracy for a given task epoch (which may vary in length), we computed the root mean square of the NPC time course between the events defining the task epoch (mean ± SEM across bootstrap estimates). In other words, we computed the area under the time course normalized by the length of the epoch. This allowed us to compare the NPC across task epochs of different lengths in an unbiased fashion because the NPC computed directly from the spike counts collected over epochs of different lengths is dependent on the length of the epoch.

We examined the possible sources of the NPC updating by computing the onsets and peaks of the time course of the NPC across the entire task. First, we smoothed the NPC time courses using a truncated Gaussians over a 250 ms window. We then compared for each time step the derivatives of the NPC time course before and after. We finally determined the onsets by maximizing the difference of the derivatives after and before the onset, and the peaks by maximizing the difference of the derivatives before and after the peak.

### Recursive neuronal elimination

To quantitatively rank the contributions of each neuron to the NPC, we developed a technique derived from machine learning (Duda et al., 2001; Guyon et al., 2002): Recursive Neuronal Elimination (RNE). RNE finds the subset of most important neurons by iteratively removing neurons that least affect the accuracy of the NPC. We first pooled all recording sessions that had at least nine trials per oculomotor behavior, for a total of 325 independently recorded neurons. We then found the neuron(s) that when removed from the population maximized the decoding accuracy across the remaining neurons. We iteratively applied this procedure to the remaining neurons, thus creating a list of neurons ranked from least to most important. The decoding accuracy was averaged across the three behaviors because the same neuronal subset was used to predict each one of them. Also, we determined the decoding accuracies using the root mean square across the task epochs that best mediate each behavior: the memory, fixation and fixation II epochs for the saccade direction, pre- and postsaccade eye position respectively.

RNE yields populations of neurons that are devoid of non-task related neurons and redundant neurons (e.g., neurons with similar tuning). It is thus ideally suited to study the dependency of prediction accuracy and population size in a principled manner that avoids using neuronal subsets defined by random pooling (Bremmer et al., 1998). As such RNE can be used to assess the sparseness of the NPC. Also, RNE is a quantitative method to address sampling bias in large neuronal populations. Finally, once an ‘optimized’ subset is defined, the accuracy of the NPC for this subset can be used to correct the accuracy of the NPC for each empirical population by scaling its average decoding accuracy to mach the one of the ‘optimized’ subset.
