# Supplementary material for: Inferring eye position from populations of lateral intraparietal neurons
Source: eLife. 2014 May 20;3:e02813. doi: 10.7554/eLife.02813 (PMC4021542; doi:10.7554/eLife.02813)
Supplement: Related texts

# Article commentary: Decision letter

**DOI:** 10.7554/eLife.02813.007

**Affiliations:** Universidad Nacional Autonoma de Mexico, Mexico

eLife posts the editorial decision letter and author response on a selection of the published articles (subject to the approval of the authors). An edited version of the letter sent to the authors after peer review is shown, indicating the substantive concerns or comments; minor concerns are not usually shown. Reviewers have the opportunity to discuss the decision before the letter is sent (see review process). Similarly, the author response typically shows only responses to the major concerns raised by the reviewers.

Thank you for sending your work entitled “Inferring eye position from populations of lateral intraparietal neurons” for consideration at eLife. Your article has been favorably evaluated by Eve Marder (Senior editor) and 3 reviewers, one of whom, Ranulfo Romo, is a member of our Board of Reviewing Editors, and we are very pleased to inform you that your work has been accepted for publication.

One of the reviewers has just two minor comments for your consideration:

First, a description of the correction for sampling bias could be given briefly in the Results, as this is just a scaling of the ensemble decoding accuracy by the performance of the optimized population, and it took me a while to find it in the Methods.

Second, both truncated Gaussian and Poisson likelihood functions are given in the Methods, but I couldn't find which was actually reported in the Results (although it is stated that they give highly correlated results).

[Editors’ note: there is not an accompanying Author response for these minor points.]
